# Modus Operandi in Sexual Assaults of Female Strangers Does Not Change Over Time

**DOI:** 10.1177/10790632221139174

**Published:** 2022-11-09

**Authors:** Eric Beauregard, Julien Chopin, Martin Andresen

**Affiliations:** 1 School of Criminology, 1763 Simon Fraser University, Burnaby, BC, Canada; 2 Terrorism, Violence and Security Institute Research Centre, 1763 Simon Fraser University and International Centre for Comparative Criminology, University of Montreal, Burnaby, BC, Canada

**Keywords:** sexual violence, modus operandi, time series, structural breaks

## Abstract

Criminological theories and widespread assumptions about crime suggest that the modus operandi involved in sexual crimes should have changed over time given various contextual changes, such as better criminological knowledge (e.g., forensic awareness) as well as improved investigative techniques (e.g., forensic evidence analysis). The aim of this study was to test whether the modus operandi patterns of individuals having committed a sexual assault against female strangers have changed over time during the period of 2003–2017. More specifically, the study has identified changes in the trends of monthly counts and (relative) participations for sexual assaults during the study period in France. The measure of participations – a concept borrowed from the field of criminal career – was used to overcome the inherent limitations associated with this type of data. Results show that despite some significant changes in the modus operandi involved in sexual crimes, overall the modus operandi patterns appear to be fairly stable over time. The findings are discussed in light of their theoretical and practical implications.

## Introduction

Over the years, research on sexual violence has evolved. From a mainly offender-focus perspective, it has slowly incorporated the victim as well. However, despite the acts themselves being important in this alchemy of crime (Felson, 1998), very few studies focused on this aspect. Most studies would include a description of the offenders’ behaviors during the crime, but it took longer for research to focus on types of offenders that, in addition to offender characteristics, would include the modus operandi. This modus operandi has now become very important in the classification of offenders (Proulx & Beauregard, 2009).

Modus operandi is a term which designates the pattern of actions and behaviors prior to, during, and following the commission of an illicit act (Hazelwood & Warren, 2003; Sutherland, 1947). By the late 1930s, modus operandi identification had become a standard procedure in criminal investigation. Although Sutherland (1947) referred to modus operandi as a technique used repeatedly and without any change to commit a crime, it became clear in the late 1980s that modus operandi could fluctuate from crime to crime. The modus operandi has been conceived as a dynamic process which needs to be adapted to situational circumstances (Cornish, 1993).

Amir’s book, *Patterns in Forcible Rape* (1971), has been known as the first sociological study on the phenomenon of rape. The study described data that came from police records of 646 victims and 1292 offenders in Philadelphia between 1958 and 1960. After establishing the background of the offender and the victim, he then focused on the crime itself: when (e.g., month, day, hour), where (e.g., residence of victim, same area of victim), how (e.g., presence of alcohol, previous record), and what (e.g., planning, use of force, humiliation, victim’s resistance).

Although Amir’s work presented a very informative and detailed picture of the crime of rape, many today disregard or overlook his work for being outdated. It is believed that the modus operandi cannot be the same as it was in the 1960s. Although most people would instinctively agree with such assumption, it has never been empirically tested. We believe that such a study is fundamental insofar as it allows us to better understand whether changes that occur over a period of time – both at the macro (e.g., routine activities, lifestyle) and micro (e.g., police techniques) levels – affect the behaviors of the individuals involved in sexual offending or not. The answer to this question, beyond contributing to a deeper understanding of sexual offending, could contribute to the debate on the methodological biases related to the study of criminal behavior with longitudinal data. Therefore, the aim of this study is to examine whether the modus operandi in sexual assault has changed over time.

### Literature Review

#### Why Modus Operandi Should Have Changed Over Time

Although to our knowledge no other researchers have examined the change – or lack thereof – in modus operandi over time, existing research has provided some indications that individuals should have adapted their crime-commission process over the years. In this context, the seminal work of Cohen and Felson (1979) aimed to understand the evolution of crime trends in the United States from the end of World War II to 1974. One of the many conclusions of this research suggested that social changes lead to a shift in opportunities which impacted criminal activities. In particular, the routine activities perspective assumed that social changes that took place in a temporal space resulted in shifts in daily activities that would then affect criminal activities. More specifically, they noted that daily routine activities “affect the location of property and personal targets in visible and accessible places at particular times, thereby influencing their risk of victimization. Furthermore, by determining their size and weight and in some cases their value, routine production activities should affect the suitability of consumer goods for illegal removal” (Cohen & Felson, 1979, p. 594). Changes in the size and weight of consumer goods, for instance, have increased the opportunities available to potential offenders and, by analogy, the modus operandi required to steal them (Cohen & Felson, 1979). In other words, time brings about social changes that affect the targets and their exposure and lead criminals to adapt their process to access them and commit their crime.

Another of these perspectives focuses on the “exit” stage of the crime-commission process (Cornish & Clarke, 2002). Offenders sometimes have to change the way they commit their crimes to avoid apprehension. This has been captured by the concept of restrictive deterrence, which refers to any strategies employed by the offender to evade detection (Gibbs, 1975). Jacobs (1996a, 1996b) expanded on this concept and proposed two distinct types: *probabilistic* (reduction of offense frequencies based on a “law of averages” mentality) and *particularistic* (reduction in offense frequencies based on tactical skills offenders use to avoid apprehension). Jacobs (1996b) distinguished further the particularistic deterrence into reactive and anticipatory dimensions. While reactive restrictive deterrence concerned the strategies used by offenders to filter or neutralize social control agents (and/or their related apparatus) before offenses are committed, anticipatory restrictive deterrence included specific strategies used by offenders to avoid detection regardless of direct contact with social control agents. As an example, in his study of crack dealers, Jacobs (1996a) identified a “perceptual shorthand” offenders used to determine whether potential buyers were narcs, focusing on the buyers’ appearance (e.g., clean vs. filthy appearance, large clothes to hide wires or bulletproof vest), verbal clues (e.g., soliciting crack improperly), and testing (e.g., forced drug use of the spot). In response to police practices, these strategies have been developed by crack dealers to help them avoiding arrest. However, the study by Jacobs (1996a) did not address whether these strategies changed the way these criminals conducted their business.

Transposing this concept to sexual assaults, Beauregard and Bouchard (2010) examined the presence of forensic awareness in serial rape events. Forensic awareness refers to an offender’s knowledge or understanding of the importance of forensic evidence (e.g., DNA, fingerprints, dental impressions) to police investigation (Davies, 1992). It can be defined as taking additional steps and adapting one’s modus operandi to hide evidence to ultimately avoid apprehension (Davies, 1992). Interestingly, Beauregard and Bouchard (2010) found that among the 222 rape events included in their study, offenders displayed some form of forensic awareness in only 55.9% of cases.^1^ In addition, they observed that offenders were not necessarily consistent in their use of forensic awareness from one event to the other. Even more important was the fact that in only a minority of rape events did offenders take precautions to destroy or clean up the one specific type of evidence – DNA – despite offenders ejaculating at the scene in 63.5% of the rape events investigated. Such findings showed that although offenders were aware of various factors that may lead to their apprehension (e.g., evidence left at the crime scene), changing their modus operandi was not automatic and represented a challenge for many of them (e.g., due to their low self-control; see General Theory of Crime by Gottfredson & Hirschi, 1990). Also, in some cases offenders will not deny the sexual encounter but will claim that it was consensual.

Another approach that could explain the need for a change in the modus operandi of individuals committing a sexual assault is the CSI effect hypothesis. The “CSI effect” takes its name from *CSI: Crime Scene Investigation*, a police television show that focuses on the use of forensic sciences to solve crimes. This television show first aired in 2000 and has been one of the top three television programs watched across America, with a reported 25 million viewers in the 2005/2006 season (Cole & Dioso-Villa, 2007). According to the original hypothesis, “watching television shows, such as *CSI*, has influenced the general public’s attitudes, expectations, and decision making related to the use of scientific evidence in jury trials” (Baskin & Sommers, 2010, p. 97). Although empirical studies assessing whether or not the CSI effect really exists have come to opposing conclusions (see Baskin & Sommers, 2010; Holmgren & Fordham, 2011; Kim et al., 2009; Schweitzer & Saks, 2007; Smith et al., 2007; Stinson et al., 2007; Tyler, 2006), one specific type of CSI effect suggested by Cole and Dioso-Villa (2007) – the *police chief’s effect* – states that crime dramas such as *CSI* are in fact educational for criminals, providing them with new strategies to learn how to avoid police detection as well as an increased sophistication in the commission of their crime. Therefore, according to this hypothesis, we should observe an increase in the use of forensic awareness strategies as well as changes in the modus operandi with the appearance of all these crime dramas.

To examine this hypothesis, Beauregard and colleagues (Beauregard & Martineau, 2014; Chopin & Beauregard, 2020b) have calculated the mean use of forensic awareness strategies for 5-year periods from prior to 1960 to 2010 in cases of sexual homicide and stranger rapists in Canada and France. Their findings showed that although the use of detection avoidance strategies began to increase significantly during the 1970s to reach a peak in the mid-1980s, the mean use of these strategies has remained relatively stable ever since, contradicting this hypothesis. Once again, these findings have suggested that despite the availability of additional knowledge for criminals, it did not lead to a significant change in how they committed their crimes.

Finally, it can be argued that recent improvements in criminal investigation may have triggered a change in the modus operandi of individuals involved in sexual crimes. For instance, in the past 20 years, the use of forensic evidence in criminal investigation has increased significantly, due in part to the technological progress achieved to analyze even microscopic pieces of evidence (Beaver, 2010; Strom & Hickman, 2010). To commit their crimes, offenders take actions that inherently have the potential to leave evidence at the crime scene and this is especially true in sexual crimes. For instance, the use of restraints may be linked to the offender by analyzing fibers recovered at the crime scene or a sexual assault victim may have been penetrated and the offender ejaculated, leaving DNA linking him to the crime. The technological progress associated with criminal investigation practices should have resulted in some changes in the offenders’ modus operandi. As an example, offenders could have started using condoms when assaulting their victims to prevent leaving DNA, or even wearing gloves to avoid leaving fingerprints at the crime scene. Yet, existing research does not seem to confirm such assumption. Some research findings may partially explain why.

Despite all these potential opportunities, unavailability of many forms of forensic evidence has been observed at crime scenes (Cole, 2010). In fact, research has shown that physical evidence is being collected in less than 10% of all cases investigated by the police (Horvath & Meesig, 1996). More specifically, research focusing on the role of forensic evidence in criminal investigations has led to three main observations. First, the police collect far more physical evidence than will be submitted for analysis and a significant amount of collected scientific evidence is of little use in many cases (Greenwood & Petersilia, 1975). Second, most forensic evidence does not lead to the identification of (a) suspect(s) – without witness testimony – and is used primarily in the interrogation of the suspect to increase the likelihood of obtaining a confession (Sanders, 1977; Simon, 1991; Wilson, 1976). Third, the main reasons why evidence is not submitted for analysis are (1) financial considerations, (2) a suspect has been arrested, and (3) perceived usefulness of the evidence type (Tilley & Ford, 1996). However, a quick look at these studies allows us to notice that they are all dated. Considerable progress has been made in the area of forensic science resulting in enhanced analytical capabilities and a reduction in the amount of evidence required for analysis. The National Institute of Justice (NIJ) has recently funded several studies to examine the analysis of forensic evidence in criminal investigation (e.g., Ritter, 2011). Why is there an interest in the use of forensic evidence in criminal investigation at this time? Because as surprising as it may seem, the best evidence currently available suggests that the collection and use of forensic evidence during the course of criminal investigations has not kept pace with advances in forensic science. Although we have the means and the expertise to collect more evidence, the reality is that very little collected evidence is actually being analyzed. This has been shown by the numbers of untested sexual assault kits (SAK)^2^ that have been discovered in police evidence room across the US: 10,000 in Los Angeles, 12,000 in Dallas, 10,500 in Detroit to name a few (Ritter, 2011). A survey conducted by the NIJ in 2007 revealed that 18% of unsolved sexual assaults that occurred between 2002 and 2007 contained forensic evidence that remained in police custody and was not submitted for analysis to a crime lab (mainly consisting of serological/biological evidence and DNA; Strom et al., 2009). Part of the problem appeared to stem from backlogs – which is different from untested evidence awaiting submission to a crime lab. NIJ defined a case as backlogged when it “has not been tested 30 days after submission to the crime laboratory” (Nelson, 2010, p. iii). Although laboratories are able to analyze more evidence than ever, more cases are submitted than can be analyzed. As mentioned, it is important to point out that recent studies have shown that the situation has improved in some jurisdictions but that in others, the same issues still persist today (e.g., Muldoon et al., 2018).

## Current Study

Similar to how our lives have been influenced by technology, crime – more specifically the way crimes are committed – has been impacted by the various technological developments, education, as well as better investigative practices. However, despite the progress achieved and the various changes that occurred over the last two decades, to our knowledge no one has examined whether the modus operandi used to commit sexual assaults has changed. Given that existing literature suggests that individuals involved in sexual crimes should have changed and adapted their modus operandi to the new reality to continue being successful and to avoid detection, the aim of this study was to test whether the modus operandi of individuals having committed a sexual assault against female strangers has changed over time. More specifically, the current study assessed any changes in modus operandi in both absolute and relative frequency in France between 2003 and 2017. The question of change in modus operandi over time is not only important for police investigation and prevention, but also for those involved in research on the crime-commission process in sexual crimes.

## Methods

### Sample

The sample used in this study comes from a national database operated by the French Ministry of Interior and included information relating to 2489 stranger (i.e., individual involved in crime and victim did not know each other at the time of the offense) rape cases, which occurred between 2003 and 2018 in France. Some of the data have been used – in whole or in part – in other studies as well (e.g., Chopin & Beauregard, 2021; Chopin et al., 2019; Chopin et al., 2021). This database includes information related to offender, victim, and crime characteristics. The data included in the database come from various sources of information. To avoid missing data, information is compiled by a team of crime analyst experts in violent crimes. Although it is still possible to have missing values as the information may not always be known, this was not the case with the variables examined in this study. For each case, the information comes from investigative reports, interview reports, medical/autopsy reports provided by pathologists, psychological reports provided by a team of forensic psychologists, and crime scene forensic reports. As is the case with most official databases used for operational purposes, information related to the coding process is not available as no inter-rater reliability measurements are performed. However, instead of using only police reports or interviews with offenders, the current study used data compiled by a team of crime analysts responsible to code each violent crime recorded on the France territory. These crime analysts have been trained on how to code each variable of the database. Given this is a database used for operational purposes, the training of the crime analysts constitutes a safeguard for the quality of the information entered in the database (unlike using only police reports). Moreover, using all the available sources of information available for each criminal event (e.g., police report, victim statement, offender interview) allows the crime analysists to conduct triangulation of information, again adding to the quality of information used in the study.

We report how we determined our sample size, all data exclusions, all manipulations, and all measures in the study. For the purpose of this study, we applied several criteria to select cases included in the research sample from the original database. First, a decision was made to exclude all cases with two or more victims (i.e., serial offenders) as several studies have shown differences in the modus operandi of serial versus nonserial offending (e.g., Chan et al., 2015). Second, we decided to exclude all cases involving child victims. Several studies highlighted differences in the modus operandi followed by offenders who sexually assaulted children and adults (Bard et al., 1987; Beauregard et al., 2012; Hayman et al., 1968; Paquette & Chopin, 2022; Spohn, 1995). There is no consistent method to operationalize what constitutes child and adult victims. Consequently, we decided to follow the guidelines provided by previous studies and therefore the current sample excluded all cases involving victims under 16 of age (Chopin & Caneppele, 2019; Leclerc et al., 2009, 2010). Third, it was decided to include only cases involving female victims. Several studies found gender differences in the modus operandi used in sexual offending (Kaufman et al., 1995). Fourth, in order to use a homogeneous sample of cases, we selected only rape cases that were operationalized as the occurrence of vaginal and/or anal penetration (not oral rape) with a penis and/or foreign objects (i.e., unanimated objects used to perform vaginal and/or anal penetration).

### Data

The descriptive statistics for the various modus operandi are presented in Tables 1 and 2. We include descriptive statistics based on both the monthly counts of each modus operandi (Table 1), and the participation rates of each modus operandi for each month (Table 2). Please note that among the variables examining the modus operandi, we have included one that account for whether the crime was solved or not. Because of the low-count nature (non-normal) of the monthly count data, the median, minimum, and maximum are shown in Table 1; however, it should be noted that the means are often similar to the values of the medians. The monthly counts of these modus operandi, and the counts of these events overall, are all decreasing from 2003 through to 2017; as such, any changes identified below are in the context of decreasing sexual violence reported to the police. It is important to note that there is “double counting” in these data because multiple modus operandi may be used in the same event, the counts for many of these modus operandi are quite low, necessitating count-based regression models. Tests for the equality of means and variances are rejected in almost all cases, such that we use negative binomial regressions in all cases for ease of comparability—when Poisson models are appropriate, their results are the same as negative binomial models.

**Table 1.** Modus Operandi, Descriptive Statistics, Average Per Month.

Modus Operandi	Median	Minimum	Maximum
Daylight	4	0	15
Solved	8	0	26
Victim targeted^3^	1	0	14
Con approach	7	0	21
Surprise	3	0	11
Blitz	2	0	15
Beat	3	0	10
Stabbing/cutting	0	0	3
Strangulation/Asphyxiation	1	0	7
No physical violence was used	1	0	16
Use of restrains	0	0	5
Weapon involved	4	0	14
Weapon used	2	0	8
Weapon brought to the scene	3	0	13
Weapon found at the scene	0	0	3
Type of weapon: Knife	4	0	13
Type of weapon: Blunt	1	0	6
Type of weapon: Firearm	0	0	3
Type of weapon: Ligature	0	0	2
Unusual act: Foreign objects inserted into the victim (vaginal/anal)	0	0	3
Unusual act: Mutilation of genitals	0	0	2
Items taken	3	0	12
Victim was intentionally released	9	0	27
Vaginal intercourse	12	0	31
Anal intercourse	4	0	16
Fellatio	5	0	15
Cunnilingus	1	0	7
Penetration with fingers	3	0	14
Masturbation	2	0	9
Fondling	5	0	21
Ejaculation on victim	0	0	7
Number of sexual acts committed	41	0	124
Acts of sexual sadism: humiliation^4^	2	0	12
Destroying evidence: Staging crime scene	1	0	5
Protecting identity	4	0	12
Acting on victim^5^	6	0	20
Number of forensic awareness strategies	16	0	53
Any semen located	6	0	17
Contact scene: Victim’s residence	2	0	10
Contact scene: Offender’s residence	0	0	2
Contact scene: Outdoors	6.5	0	19
Crime scene: Victim’s residence	3	0	11
Crime scene: Offender’s residence	1	0	5
Crime scene: Outdoors	6	0	19
Release scene: Victim’s residence	3	0	10
Release: Offender’s residence	0	0	5
Release: Outdoors	5	0	17
The victim was injured	10	0	29
Verbal resistance from victim	5	0	17
Physical resistance from victim	3	0	13

**Table 2.** Modus Operandi, Descriptive Statistics, Average Participations Per Month.

Modus Operandi	Median	Mean	Maximum
Daylight	1.79	1.85	4.21
Solved	3.84	3.87	7.61
Victim targeted	0.63	0.87	3.92
Con approach	2.98	2.96	7.27
Surprise	1.64	1.74	4.08
Blitz	1.11	1.18	9.09
Beat	1.31	1.43	9.09
Stabbing/cutting	0	0.14	2.04
Strangulation/Asphyxiation	0.62	0.74	4.08
No physical violence was used	0.58	1	5.39
Use of restrains	0	0.31	2.56
Weapon involved	1.99	2	4.08
Weapon used	0.8	0.85	2.98
Weapon brought to the scene	1.67	1.61	3.47
Weapon found at the scene	0	0.18	2.04
Type of weapon: Knife	1.77	1.8	4.08
Type of weapon: Blunt	0.22	0.37	2.41
Type of weapon: Firearm	0	0.09	1.12
Type of weapon: Ligature	0	0.04	0.61
Unusual act: Foreign objects inserted into the victim (vaginal/anal)	0	0.12	1.06
Unusual act: Mutilation of genitals	0	0.06	1.39
Items taken	1.54	1.5	3.95
Victim was intentionally released	4.24	4.21	6.21
Vaginal intercourse	5.42	5.43	9.09
Anal intercourse	1.79	1.77	6
Fellatio	2.45	2.39	4.83
Cunnilingus	0.31	0.39	2.11
Penetration with fingers	1.54	1.61	4.44
Masturbation	0.82	0.86	2.86
Fondling	2.48	2.45	9.09
Ejaculation on victim	0	0.34	2.07
Number of sexual acts committed	18.45	18.06	24.49
Acts of sexual sadism humiliation	0.96	0.99	2.9
Destroying evidence: Staging crime scene	0.44	0.5	2.11
Protecting identity	1.69	1.63	4
Acting on victim	2.65	2.7	5.13
Number of forensic awareness strategies	7.04	7.05	13.5
Any semen located	2.56	2.64	7.27
Contact scene: Victim’s residence	1.17	1.2	3.54
Contact scene: Offender’s residence	0	0.05	1.09
Contact scene: Outdoors	3.08	3.12	9.09
Crime scene: Victim’s residence	1.38	1.46	4
Crime scene: Offender’s residence	0.22	0.32	2.17
Crime scene: Outdoors	2.57	2.65	9.09
Release scene: Victim’s residence	1.28	1.39	4
Release: Offender’s residence	0	0.24	2.17
Release: Outdoors	2.5	2.55	9.09
The victim was injured	5.06	4.87	9.09
Verbal resistance from victim	2.21	2.35	9.09
Physical resistance from victim	1.46	1.51	7.41

*Note.* Minimums are all zero.

The descriptive statistics for the percentage of modus operandi participations are presented in Table 2. As noted above, the modus operandi (MO) are shown using the concept of participations, borrowed from the co-offending literature (Andresen & Felson, 2012a, 2012b). With the presence of 50 possible MOs for each offence – which have been selected based on previous studies of the crime-commission process analysis of interpersonal assaults (see e.g., Cale et al., 2021; Chopin & Beauregard, 2020a) – each offence has 50 possible participations. For example, one sexual offence could include daylight, surprise, and vaginal intercourse (3 participations in the offence) whereas another sexual offence could include blitz, fellatio, vaginal intercourse, and protecting identity (4 participations in the offence).

As such, the dependent variable represents a percentage of total participations in each month for each MO. This allows us to identify changes in the nature of sexual offences over time. For example, the frequency of sexual offences may be decreasing over time (the case for our data) but the nature of those offences may be changing over time because of changes to police tactics (forensics, for example) or to the law. Because of this, offenders may choose to “participate” in fewer of one MO and more of another; and if one measured the frequency of a particular MO that is increasing relatively, it may still be decreasing because there are fewer offences. Using the percentage of total participations account for this type of change. The means, standard deviations, and medians reported in Table 2. These values range from zero to one hundred (the percentage multiplied by 100). All but two of the MOs are dichotomous in nature (Number of sexual acts committed and Number of forensic awareness strategies). Consequently, most of the percentages are relatively low.

### Statistical Analyses

We analyzed the absolute frequency of various modus operandi by considering the monthly counts of the various modus operandi in sexual assaults in France, 2003 to 2017. However, because the number of sexual assault events have been decreasing through these years, as most property and violent crimes have been since the early 1990s worldwide (Farrell et al., 2011), any decreases in frequency may simply be due to the decrease in sexual assaults, overall. It is possible that changes in the modus operandi of sex offenders may be identified through different magnitudes of decreasing trends (some modus operandi may decrease more or less than others, compared to the number of events overall), but such differences may be difficult to identify, particularly with relatively low count events, on a monthly basis. We are addressing this issue, and contributing to the literature, through the use of an additional measurement of modus operandi, the participation. Each event has the potential to have multiple modus operandi or characteristics: the contact site, the crime site, the presence of a weapon, acts committed, and so on. As such, an offender can “participate” in multiple modus operandi within a single event. This is similar to the concept of participations in the co-offending literature in that one criminal event may occur, but there are as many participations in that event as there are offenders (Andresen & Felson, 2012a; 2012b). By measuring participations, specifically the percentage of participations in sexual assaults with a particular modus operandi (forensic awareness, for example), we can easily identify changes in the modus operandi of offenders easily even when the overall frequency of events is decreasing over time. In the analyses below, we have identified changes in the trends of monthly counts and (relative) participations for sexual assaults in France between 2003 and 2017.

Structural break analyses, sometimes referred to as interrupted times series, are used to identify changes that may have occurred in the trends of time series. In the analyses below, we use a structural break methodology in conjunction with an endogenous Chow (1960) testing methodology. Structural break methods are used increasingly in the criminological literature to evaluate policy changes, crime reduction programs, or simply changes in behavior (Hodgkinson & Andresen, 2020; Hodgkinson et al., 2018; Mohler et al., 2020; Piehl et al., 1999; Reid & Andresen, 2014). Structural break analyses may be undertaken using either exogenous or endogenous testing methodologies. Exogenous testing methodologies are used when an event (e.g., policy change) occurs at a specific date and its effect is evaluated. Endogenous testing methodologies are used when a specific date for (expected) change is not known or does not want to be assumed within the analyses. This latter testing methodology, used in the analyses below, is more intensive because one must sequentially test all possible structural breaks. As such, this methodology does not impose a break in the data because of a known events but searches for the presence of a structural break. If a structural break in the data has occurred (resulting from a change in modus operandi, for example), that structural break and its timing is identified through statistical significance and can then be compared to known events to identify potential causal factors. Such a methodology is preferable to exogenous testing methodologies that impose a specific date for a structural break. This is because a structural break may be identified as statistically significant even when the temporal sequence is incorrect: a structural break may have occurred years before and unrelated to a policy change but emerge as statistically significant.

In the current context we wish to investigate if different modus operandi change at expected times and with expected grouping. As such, we use a sequential Chow (1960) test to endogenously search for a change in the trends of the modus operandi in sexual assault

$${MO}_{t}=\alpha +\beta_{1}Month+\beta_{2}{Month}^{2}+{\beta_{3}Days+\beta_{4}Trend+\gamma }_{1}Break+\gamma_{2}BreakTrend+\varepsilon_{t},$$

where *MO* is the modus operandi type (monthly count and participations), *Month* and *Month*^2^ are included to account for the seasonal component in crime data (Andresen & Hodgkinson, 2018; Breetzke & Cohn, 2012; Cohn & Rotton, 2000; Farrell & Pease, 1994; Linning et al., 2017), *Days* is the number of days in each month to account for any differences in counts because of there being more days in some months than others, and *Trend* accounts for the overall trend in the data that is decreasing, consistent with the international crime drop literature (Farrell et al., 2011). Each modus operandi is analyzed separately, such that each modus operandi is given equal weight, rather than imposing a pre-existing weighting system.

The structural break variables (*Break* and *BreakTrend*) allow us to identify any changes in the counts of the presence of the different modus operandi. *Break* captures any immediate or sudden change in the data series, whereas *BreakTrend* captures any changes in the trend of the data series. With 15 years of data, there are a total of 180 observations. However, in order to account for new trends to emerge over time, we exclude the first and last year of the data series to be able to experience a structural break, leaving 156 potential sequential break points to identify.

The actual structural breaks are determined at the time period that has the greatest value t-statistics for the break variables using robust standard errors (heteroskedastic and autocorrelation consistent). Due to the nature of the data shown in Table 1, monthly counts, we use negative binomial regression models to identify changes in trend in all of those cases. The results from negative binomial regressions can be interpreted in a similar way as linear regressions. That is, for each additional month, the number of participations for a given modus operandi changes approximately by B%. For the percentage of participations (continuous variables), we use generalized least squared model in all cases using robust standard errors (heteroskedastic and autocorrelation consistent). All estimation for the sequential Chow tests is undertaken using R: A language and environment for statistical computing, version 3.5.3 (Team R Core, 2019). Ethical approval was obtained to conduct this research from the Institutional Review Board of the authors’ institution. No financial support was received to conduct the research. No financial interests are involved in the current study.

## Results

The statistical output for the structural break tests is shown in Tables 3 and 4. It is important to not only consider the statistical significance of the results (all results in Table 3 are statistically significant), but the magnitude of the results. Specifically, it is critical for us to consider the magnitude of any changes because the goal of our analyses is to identify *changes* in the modus operandi of offenders. As such, the important comparison is magnitude of the trend and the post-break trend.

**Table 3.** Structural Break Tests, Negative Binomial Regression Results, Counts.

Modus Operandi	Month Break	Year Break	Trend	Break	Break Trend	Post-break Trend
Daylight	Aug	2016	−0.007	0.443	−0.137	−0.006
Solved	Sep	2014	−0.005	0.407	−0.041	−0.020
Victim targeted	Jul	2005	−0.002	−0.527	−0.018	−0.016
Con approach	Sep	2014	−0.006	0.410	−0.044	−0.033
Surprise	Apr	2011	0.002	0.281	−0.019	−0.216
Blitz	Apr	2006	0.005	−0.382	−0.021	−0.015
Beat	Mar	2010	−0.002	0.417	−0.014	−0.020
Stabbing/cutting	Jul	2015	−0.003	−3.421	0.143	−0.018
Strangulation/Asphyxiation	Jun	2011	−0.001	0.456	−0.020	−0.019
No physical violence was used	Jun	2006	0.012	−0.552	−0.046	−0.019
Use of restrains	Feb	2017	−0.014	30.02	−27.67	−0.016
Weapon involved	Sep	2012	−0.005	0.285	−0.019	−0.019
Weapon used	Jul	2011	−0.001	0.600	−0.026	−0.016
Weapon brought to the scene	Apr	2012	−0.006	0.395	−0.019	−0.019
Weapon found at the scene	Aug	2015	−0.003	−3.447	0.134	−0.021
Type of weapon: Knife	Apr	2012	−0.006	0.322	−0.016	−0.020
Type of weapon: Blunt	Nov	2004	0.058	−0.845	−0.064	−0.020
Type of weapon: Firearm	Sep	2016	−0.001	25.72	−24.11	−0.017
Type of weapon: Ligature	Jul	2015	−0.016	21.79	−17.30	−0.018
Unusual act: Foreign objects inserted into the victim (vaginal/anal)	Nov	2016	−0.012	25.43	−23.27	−0.021
Unusual act: Mutilation of genitals	Aug	2016	−0.019	−33.30	0.021	−0.021
Items taken	Oct	2010	−0.009	0.654	−0.010	−0.027
Victim was intentionally released	Sep	2014	−0.006	0.211	−0.033	−0.019
Vaginal intercourse	May	2011	−0.006	0.262	−0.013	−0.027
Anal intercourse	Aug	2010	−0.010	0.586	−0.011	−0.022
Fellatio	Jun	2010	−0.006	0.604	−0.014	−0.025
Cunnilingus	May	2008	−0.010	1.745	−0.206	−0.022
Penetration with fingers	Jul	2011	−0.008	0.368	−0.011	−0.024
Masturbation	Jul	2015	−0.008	−1.146	0.038	−0.029
Fondling	Jul	2015	−0.008	−0.629	0.015	−0.025
Ejaculation on victim	Sep	2016	−0.016	−5.808	0.394	−0.030
Number of sexual acts committed	Jun	2010	−0.008	0.438	−0.010	−0.031
Acts of sexual sadism humiliation	Sep	2012	−0.012	0.406	−0.017	−0.047
Destroying evidence: Staging crime scene	Jul	2011	−0.008	0.874	−0.019	−0.050
Protecting identity	Aug	2010	−0.008	0.509	−0.013	−0.039
Acting on victim	Dec	2012	−0.007	0.483	−0.018	−0.046
Number of forensic awareness strategies	Jan	2013	−0.007	0.638	−0.024	0.140
Any semen located	Jun	2010	−0.008	0.500	−0.011	−17.317
Contact scene: Victim’s residence	Jul	2011	−0.007	0.479	−0.015	0.029
Contact scene: Offender’s residence	Aug	2015	0.008	17.33	−16.25	0.006
Contact scene: Outdoors	Jun	2010	−0.005	0.511	−0.013	0.132
Crime scene: Victim’s residence	Jun	2011	−0.006	0.461	−0.015	−16.240
Crime scene: Offender’s residence	Oct	2015	−0.004	23.53	−23.28	−23.283
Crime scene: Outdoors	Jun	2010	−0.009	0.543	−0.007	−23.984
Release scene: Victim’s residence	Dec	2012	−0.005	0.412	−0.025	−0.144
Release: Offender’s residence	Oct	2015	−0.002	24.36	−23.98	0.002
Release: Outdoors	Jun	2010	−0.007	0.524	−0.013	−24.109
The victim was injured	Jun	2010	−0.001	0.383	−0.016	0.378
Verbal resistance from victim	Oct	2014	−0.005	0.480	−0.041	−23.284
Physical resistance from victim	Jul	2010	−0.010	0.368	−0.009	−27.681

*Note.* All trends and breaks are statistically significant at the 5% level.

**Table 4.** Structural Break Tests, GLS Results, Participations.

Modus Operandi	Month Break	Year Break	Trend	Break	Break Trend	Post-break Trend
Type of weapon: Ligature	Mar	2004	0.015	−0.147*	−0.015	0.000
Crime scene: Victim’s residence	May	2004	−0.012	0.496*	0.012	0.000
Use of restrains	Jul	2004	−0.040*	0.317*	0.038*	−0.002
Any semen located	Jul	2004	0.053*	−0.403	−0.054*	−0.001
Type of weapon: Blunt	Aug	2004	0.021*	−0.277*	−0.019*	0.002
No physical violence was used	Apr	2005	0.046*	−0.778*	−0.063*	−0.017
Crime scene: Outdoors	May	2005	0.084*	−1.003*	−0.046*	0.038
Items taken	Jun	2005	−0.032*	0.491*	0.032*	0.000
Strangulation/Asphyxiation	Aug	2005	−0.012*	0.516*	0.012*	0.000
Release scene: Victim’s residence	Sep	2005	0.017*	−0.068	−0.017*	0.000
Ejaculation on victim	Oct	2005	−0.019*	0.306*	0.015*	−0.004
Blitz	Dec	2005	0.020*	−0.941*	−0.024*	−0.004
Weapon involved	Jan	2005	−0.016*	0.325*	0.017*	0.001
Weapon used	Feb	2005	−0.027*	0.756*	0.027*	0.000
Penetration with fingers	Apr	2006	0.006	−0.420*	−0.004	0.002
Type of weapon: Knife	May	2006	−0.013*	0.323*	0.014*	0.001
Contact scene: Outdoors	May	2006	−0.010	0.832*	0.009	−0.001
Surprise	Jun	2006	−0.015*	0.859*	0.019*	0.004
The victim was injured	Jun	2006	−0.022*	1.875*	0.032*	0.010
Unusual act: Mutilation of genitals	Mar	2007	0.003	−0.146*	−0.003	0.000
Release: Outdoors	May	2008	0.016*	−0.647*	−0.014*	0.002
Con approach	Aug	2008	0.018*	−0.700*	−0.019*	−0.001
Vaginal intercourse	May	2009	0.009*	−0.388*	−0.009*	0.000
Fellatio	Mar	2010	0.002	0.763*	−0.014*	−0.012
Victim targeted	May	2011	−0.023*	0.501*	0.018*	−0.005
Destroying evidence: Staging crime scene	Jun	2011	−0.002*	0.354*	−0.004	−0.006
Weapon brought to the scene	Apr	2012	−0.002	0.595*	−0.008	−0.010
Protecting identity	Sep	2012	−0.001	0.522*	−0.017*	−0.018
Number of forensic awareness strategies	Jan	2013	−0.007	3.609*	−0.063*	−0.070
Solved	Jan	2014	0.002	1.468*	−0.055*	−0.053
Victim was intentionally released	Nov	2014	0.002	0.665*	−0.045*	−0.043
Fondling	Feb	2015	−0.005*	−1.275*	0.094*	0.089
Number of sexual acts committed	May	2015	−0.012*	−1.871*	0.095	0.083
Crime scene: Offender’s residence	May	2015	−0.000	0.397*	−0.032*	−0.032
Masturbation	Jul	2015	−0.007*	−0.589*	0.037*	0.030
Weapon found at the scene	Aug	2015	0.001	−0.404*	0.023*	0.024
Stabbing/cutting	Sep	2015	−0.000	−0.276*	0.027*	0.027
Acts of sexual sadism humiliation	Sep	2015	−0.005*	0.731*	−0.038*	−0.043
Acting on victim	Sep	2015	0.001	0.699*	−0.031*	−0.030
Contact scene: Offender’s residence	Oct	2015	0.001*	−0.111*	−0.001*	0.000
Physical resistance from victim	Oct	2015	−0.003*	−1.054*	0.061*	0.058
Daylight	Jan	2016	0.001	1.367*	−0.102*	−0.101
Contact scene: Victim’s residence	Jun	2016	−0.001	0.481*	−0.052*	−0.053
Type of weapon: Firearm	Sep	2016	0.001*	0.146*	−0.024*	−0.023
Cunnilingus	Sep	2016	−0.003	0.811*	−0.075*	−0.078
Unusual act: Foreign objects inserted into the victim (vaginal/anal)	Nov	2016	−0.001*	0.229*	−0.031*	−0.032
Release: Offender’s residence	Nov	2016	0.001*	−0.259*	−0.006*	−0.005
Beat	Dec	2016	0.005*	−2.348*	0.404*	0.409
Verbal resistance from victim	Most often positive trend, but no significant change					
Anal intercourse	No statistically significant trend or change					

*Note.* * statistically significant at the 5% level.

The results of the structural break tests of the monthly counts are reported in Table 3. These results include the month and year of the structural break for each modus operandi, as well as the value for the initial trend, the value of the break variable, the value for the break-trend variable, and the overall post-break trend (the sum of the overall trend and the break trend). Though there are variations, discussed further below, the most common result is an overall decreasing trend with a structural break that leads to an initial increase in the counts of the modus operandi followed by subsequent decreases in counts: break-trend variables are either negative (the trend decreases faster) or is positive but of a lesser magnitude than the original negative trend (the trend is still decreasing but at a lesser rate).

Though there are some individual results of interest, discussed below, there are a number of modus operandi that change at similar times that are of interest here. For example, summer 2010 shows a number of changes for 10 modus operandi. Overall, the modus operandi have decreasing trends overall, have a significant (but moderate value) jump/increase in summer 2010, followed by subsequent decreasing trends. The interesting combination of changes in modus operandi here for the contact scene, crime scene, and release all with changes for being outdoors. Moreover, there is an initial increase, followed by a decrease for the victim being injured, physical resistance, and protecting identity. Of course, the first two having (any similar) change at the same time would be expected.

The next “set” of modus operandi changes that occurred at the same time was summer 2011. Similar to the previous set of results, there is an overall decreasing trend with a significant (but moderate value) jump/increase in summer 2011, followed by subsequent decreasing trends. This set of modus operandi involve the victim’s residence (contact and crime scene), the destruction of evidence, a weapon and increases in some forms of violence. The following spring (April 2012) saw similar changes in both a weapon being brought to the scene with that weapon being a knife. This continued September 2012. And fall 2014 saw similar changes in solved crimes, the victim being released intentionally, and verbal resistance from the victim.

Summer and fall 2015 exhibits an interesting (and different) set of results. In particular, with a similar pattern as above, the offender’s residence is implicated for the contact, crime, and release scene. Perhaps most interesting are the immediate drops and subsequent increases in stabbing/cutting, masturbation, fondling, and weapon found at the scene, with the first and last modus operandi being closely linked. Though later in the data series (2016), this is also the case for mutilation of genitals and ejaculation on victim.

Turning to the results for the participations, Table 4, there are notable differences from the monthly counts, highlighting the use of different measurements of modus operandi. The first aspect of these results to note is that not all of the results are statistically significant. There are also differences in the qualitative results with regard to trends, both before and after the identified break month/year. With the monthly counts, only a small number of modus operandi exhibited positive trends post-break, but this is not the case with participations.

During spring/summer 2004, the structural breaks led to post-break trend decreases in the use of restraints and the presence of semen. The use of ligatures and blunt weapons also showed statistically significant decreases, but the magnitude of these changes was not enough to impact the overall trend of the post-break trends. Spring/summer 2005 had structural breaks indicating decreases in “non-violence”, outdoor crime scenes, and the release scene being the victim’s residence. However, only the increase presence of violence led to a change in the direction of the post-break trend. Spring/summer 2006 exhibits increases in the post-break trend of the use of a knife, surprise, and injured victims.

The next “set” of changes to modus operandi occurred in spring/summer/fall of 2015—changes occurred in the intervening years, but in a relatively isolated manner. In 2015, there were overall decreases in the use of the offender’s residence, acting on the victim, and acts of sadism—the latter, however, did exhibit a notable jump at the time of a break before it subsequently decreased. But there were also increases in weapons, stabbing/cutting, and the presence of physical resistance that led to increasing post-break trends. There are also a number of changes in 2016, but all but one of those modus operandi changes led to decreasing post-break trends. Lastly, verbal, resistance and anal intercourse had no significant changes in participations.

Overall, the results show that there are changes in the *counts* of modus operandi but not the *participations* in the modus operandi because participations better account for the overall decrease in sexual assaults reported to the police. There are statistically significant changes for participations as well, but the magnitude of those changes are not large enough to exhibit much, if any, change over time.

## Discussion and Conclusion

This study tests whether the modus operandi of individuals having committed a sexual assault on female strangers has changed over time. More specifically, it examines any changes in modus operandi in both absolute and relative frequency. In order to avoid the potential biases associated with a decrease in the number of sexual assaults being committed, an additional measure of modus operandi – the participation – has been used. Similar to the concept of participations in the co-offending literature (Andresen & Felson, 2012a; 2012b), by measuring specifically the percentage of participations in a specific modus operandi behavior, we can better identify changes in the modus operandi of offenders even when the overall frequency of events is decreasing over time.

As shown in the literature review, there is no single theory explaining the change – or lack thereof – in the modus operandi of criminals, let alone individuals who have committed sexual crimes. Instead, when drawing from various sources in criminology, there seems to be a consensus congruent with most people’s expectations that the modus operandi of criminals should change and adapt to the new reality, at least to avoid police detection. Whether it is for social changes (Cohen & Felson, 1979), restrictive deterrence (Jacobs, 1996a; 1996b), the CSI hypothesis (Cole & Dioso-Villa, 2007), or even due to the improvement in criminal investigation practices (e.g., Beaver, 2010), all imply or directly suggest that the modus operandi of a criminal should have changed over time. Yet, our findings suggest the opposite.

When looking at the count of participations, although the trend is going down for almost all modus operandi behaviors, this seems to be mainly due to the (reported) sexual assaults decreasing. When considering the participations, there is much less going on. Although it may seem like there are several statistically significant changes, they are all low in magnitude. In other words, our findings are showing that – at least for the period under study – the overall modus operandi involved in sexual crimes against female strangers is quite stable, even more so for the rare modus operandi behavior.

Although these findings may appear counter-intuitive based on the literature, some empirical studies have provided some potential explanation. For instance, in the study of Beauregard and Bouchard (2010) on the use of forensic awareness strategies in serial sexual assaults, interviewed offenders identified four main reasons why they had not used such strategies to evade detection. Some offenders reported that they did not even think about forensic awareness during the crime. As the crime was not planned, the majority mentioned that they did not think about this or that the acts were spontaneous, out of control. Some even mentioned that contrary to what we expect, they were not aware of the various risks associated with forensic evidence left at the crime. For other offenders, their inaction was mainly due to their mental state at the time of the crime. Thus, offenders explained that in addition to being intoxicated, they were too confused, or even too much angry to think about this. Beauregard and Bouchard (2010) identified a third theme that they labeled “irrational thinking”, designating these offenders who erroneously believed that if the victims consented or that as there was no penetration, no semen could be found. Finally, a few offenders explained that forensic awareness strategies were not necessary as an alternative was used (e.g., manipulation, ruse, or bribes to make sure the victims did not report the crime).

Such interpretation is congruent with the findings from Ha and Beauregard (2016), who tested the main component of the General Theory of Crime (Gottfredson & Hirschi, 1990) – low self-control – in a sample of individuals who had committed repeated sexual assaults. Results show support for the General Theory of Crime, low self-control being a significant predictor of offence behaviors that correspond closely to elements of the personality trait identified by the theory. Specifically, the results show that the impact of low self-control is least pronounced on victim selection characteristics and of greatest effect on characteristics contained in the later stages of the temporal crime sequence (e.g., exit stage to avoid detection). Thus, offenders lower in self-control exhibit behaviors during various stages of the sexual offence that are impulsive, risky, insensitive, short-sighted, physical, and aggressive, all of which correspond to the theoretically defined personality trait of low self-control.

When looking closely at the results one could argue that notable increases around 2005/2006 are observed, more specifically as to the use of a surprise approach, the intentional use of a weapon, and victim injured in sexual crimes against female strangers. Again, these are all small changes. More importantly, when looking at the context in France during this time, it was not possible to link such changes to specific events taking place at that time. Regarding the demonstration from Cohen and Felson (1979) linking social evolution, target changes, and criminal behavior adaptation, our results are not in the same direction. Several hypotheses can be formulated to explain this contrast. First, we might assume a certain timelessness in the sexual crime parameters over time. Unlike predatory property crimes, which see the evolution of targets in size, weights, and attractiveness (e.g., consumer goods, appliances, vehicles, etc.) requiring an adaptation of criminal processes to gain access to them (Hodgkinson et al., 2022), sexual crimes are part of a very different dynamic. In interpersonal crimes such as sexual offenses, targets are individuals who are in a vulnerable situation. Thus, over any period of time, it is not far-fetched to consider that sexual assault ‘targets’ (i.e., victims) do not change. This lack of evolution suddenly makes the assumption that criminal behavior may be stable over time relatively rational. The work of Aebi and his colleagues (Aebi, 2004, 2009; Aebi et al., 2002, 2014; Aebi & Linde, 2012) examining the evolution of European crime trends over time shows that interpersonal crimes and especially sexual crimes are quite stable over time in contrast to property and drug crimes. Such findings reinforce the idea that the opportunities to commit sexual assault have not changed over time either. However, it is important to emphasize the fact that our findings may not necessarily be generalized to all sexual crimes, as some specific sexual crimes (e.g., cyber sexual crimes) may have changed over the years due to recent technological developments. The second hypothesis is more methodologically based and would suggest that the time period studied in this research is not significant enough (i.e., 15 years, vs. 27 years for Cohen and Felson’s study, 1979) to capture the impacts of social change on criminal behavior.

These findings present some practical and theoretical implications. On the practical level, it is reassuring to observe that despite the proliferation of TV shows and discussion in the mass media of the various techniques involved in the analysis of forensic evidence, most offenders are not influenced by this knowledge when committing their crimes. In fact, even if some offenders are aware of this knowledge, they are likely to forget or to be distracted during the crime-commission process, neglecting to take precautions (e.g., Beauregard & Bouchard, 2010). Contrary to some specific forms of crime where technology changed the way to proceed (e.g., check fraud, bank robbery), sexual assaults are still being committed in the same way as they were when Amir (1971) first published his study. Although some variations may appear year after year, there seems to be an overall stability in the way criminals are committing sexual assaults – at least for sexual crimes involving female strangers. Investigators do not need to worry about criminals adapting their modus operandi and changing the way they commit their crime to avoid detection.

Such findings could also be useful for clinicians working with individuals who have committed sexual assaults against female strangers. More specifically, knowing that patterns of modus operandi appear to be mainly stable can inform the offense chains or their offending pathways (Proulx et al., 2014). This information can be used in the classification of these individuals (i.e., typologies), which in turn may help in the identification of specific treatment targets, such as anger management and deviant sexual preferences.

On the theoretical level, these findings are showing that it does not matter how old data on the crime-commission process are. This is important for researchers who study the modus operandi and the crime-commission process of individuals who have committed sexual crimes. This means that there is no empirical justification to limit a sample based on the years the crimes have been committed or worse, to not use (or limit) a specific dataset based on when it was collected. Moreover, these results are suggesting that what was found in the past on the modus operandi involved in sexual crimes might still be accurate today, despite the years that have passed. Researchers in the field could then go back to the pioneer work of researchers such as Amir and use their findings to better understand how and why offenders commit their crimes the way they do.

Although informative and innovative, this study is not without limitations. The most important limitation is definitely the time period that we were able to use and run the analyses. Despite having more than 40 years of data, the earlier years do not have similar number of observations, which made it difficult to include them in the analyses. We had to restrict the sample. Doing so made the testing of the CSI effect – particularly the impact of such TV shows that aired in the early 2000s – difficult. We hope that future studies could attempt to replicate these results by including a longer time period, especially one that would include at least the 1990s. Also, the study is based on police data, which depend largely on the crimes being reported and the information provided by the victims during the investigation. It is therefore possible that the current findings are not generalizable to all sexual assaults against female strangers and that some modus operandi indicators may have been missing. Similarly, it is important to mention that the current findings exclude serial sexual assaults. Considering that it has been demonstrated that the modus operandi used in serial offending is different from single-victim crimes (e.g., Chan et al., 2015), we cannot exclude the possibility that examining serial offending only would have produced different findings. Another limitation is that considering the data come from an operational database, it was not possible to conduct reliability and validity tests. Moreover, we should not exclude the fact that the crimes examined in the current study are only those crimes reported to the police. Therefore, it is possible that crimes using different strategies have been committed but not reported to the authorities. There is always a possibility that some offenders have found a new crime strategy that increases their chances of avoiding police detection. Future studies need to use a different source of information, such as victimization surveys to see if victims who did not report their sexual assault have had a different experience than those who disclosed the assault to the police. Moreover, it is important to address the fact that we are not describing the modus operandi of particular perpetrators over time; instead we are describing modus operandi patterns in cases over time. Thus, our cases involve a novel perpetrator who was detected only once, but hidden variations in their specific modus operandi may have been present as some would be first-time perpetrators might have been committing many sexual assaults without being detected. Finally, we are aware that some modus operandi behaviors are more difficult to observe, especially for the police. For instance, some offenders may use forensic awareness strategies that may go undetected. If the offender does not share his strategy with the police, it is very likely that a particular behavior may go unnoticed (e.g., the offender did something to hide his identify). Future studies should use a more qualitative approach to question offenders about the various strategies used to avoid police detection.

Criminals have often been described as capable of adapting to different circumstances and to change their behavior based on the situation. The current study tested whether patterns in the modus operandi involved in sexual crimes changed over time. Although some theoretical and empirical knowledge seemed to suggest that their behavior should have changed over the years, our findings have shown the opposite. Should we really be surprised? Numbers are showing that approximately only 8% of sexual assaults are reported to the police and that of these, only a small fraction will lead to the accusation and conviction of the person responsible for this crime (see Spohn & Tellis, 2014). The fact that only a minority of sexual crimes are detected and reported to the police may explain why the modus operandi involved in these crimes has remained relatively stable over time.

## Notes

1. Forensic awareness was considered to be evident during a rape if the offender did at least one of the following: concealed his identity, wore gloves, cleaned up semen, prevented his face from being seen, lied about his name, wore a condom, did not ejaculate in or on the victim, made the victim comb her pubic hair after the assault, and/or made the victim shower after the assault.

2. Introduced in the 1960s, a SAK “is a box or envelope used to collect and store biological evidence from the victim of an alleged sexual assault. Evidence in a SAK can include vaginal, oral or anal swabs that may yield the perpetrator’s DNA” (Ritter, 2011, p. 1).

3. This means that the victim was not selected due to an opportunity but was specifically targeted for to certain characteristics (i.e., physical or psychological).

4. This can be either verbal (e.g., insulting the victim) or physical humiliation (e.g., urinate on the victim), but both aim to degrade and bring down the victim.

5. Such precaution includes threatening the victim not to report, administrating a drug to the victim, tying up the victim, blocking access in and out of doors or windows, and killing the victim for the purpose of eliminating the witness.
